# Selectivity in early prosocial behavior

**DOI:** 10.3389/fpsyg.2014.00836

**Published:** 2014-07-29

**Authors:** Valerie A. Kuhlmeier, Kristen A. Dunfield, Amy C. O’Neill

**Affiliations:** ^1^Department of Psychology, Queen’s UniversityKingston, ON, Canada; ^2^Department of Psychology, Concordia UniversityMontreal, QC, Canada

**Keywords:** prosocial behavior, reciprocity, partner choice, social evaluation, cooperation

## Abstract

Prosocial behavior requires expenditure of personal resources for the benefit of others, a fact that creates a “problem” when considering the evolution of prosociality. Models that address this problem have been developed, with emphasis typically placed on reciprocity. One model considers the advantages of being selective in terms of one’s allocation of prosocial behavior so as to improve the chance that one will be benefitted in return. In this review paper, we first summarize this “partner choice” model and then focus on prosocial development in the preschool years, where we make the case for selective partner choice in early instances of human prosocial behavior.

## INTRODUCTION

Human social behavior is frequently marked by actions that are generated on behalf of others. As adults, we show great flexibility in our production of prosocial acts and readily identify these behaviors in others. Yet, the fields of anthropology, biology, economics, philosophy, and psychology have long noted that widespread engagement in prosociality is somewhat surprising, as it requires expenditure of personal resources for the benefit of others, including others with whom we share no appreciable genetic relatedness (e.g., Axelrod, 1984).

Seminal models that address this “problem” of prosociality have been developed, with emphasis typically placed on reciprocity. One model, described more fully below, considers the advantages of being selective in terms of one’s allocation of prosocial behavior so as to improve the chance that one will be benefitted in return. In this review paper, we first present the fundamental aspects of this “partner choice” model (for a more detailed discussion, see Roberts, 1998; Bshary and Noë, 2003; Baumard et al., 2013). Then, emphasis is placed on prosocial development in infancy and the preschool years, and we make the case for selective partner choice in early instances of human prosocial behavior.

This review article primarily focuses on the first 5 years of life, an age range that has received much attention in recent studies of prosocial development (for review, see Eisenberg et al., in press). We define prosocial behavior as the intervening, beneficial actions that are preceded by the direct observation or inference of another’s negative state (e.g., Dunfield and Kuhlmeier, 2013; Warneken, 2013a; Dunfield, 2014). These negative states can include instrumental need (i.e., an individual is having difficulty completing a goal-directed behavior such as retrieving an out of reach object, and a person can intervene by *helping*), material desire (i.e., an individual does not have a desired resource, and a person can intervene by *sharing*), and emotional distress (i.e., an individual is experiencing a negative emotional state, and a person can intervene by *comforting*). Each type of prosocial behavior has been examined and documented in early childhood (e.g., Zahn-Waxler et al., 1992; Hay et al., 1999; Warneken and Tomasello, 2006; Brownell et al., 2009; Dunfield et al., 2011; Dunfield and Kuhlmeier, 2013; Paulus et al., 2013; Paulus, 2014). Selective helping and sharing have been the focus of most of the research work to date on selective prosocial behavior and thus will be emphasized here; however, “sharing” in some of these instances is considered broadly to include the adult-encouraged distribution of resources to those who have none, not just the spontaneous response to others’ lack of resources.

## RECIPROCITY AND PARTNER CHOICE

Reciprocity solves the “problem” of prosociality because an individual’s investment can be repaid. It is a mutually beneficial, universal feature of human social organization (e.g., Brown, 1991), appearing as both direct reciprocity (e.g., B helps A in return for A having helped B; Trivers, 1971) and indirect reciprocity (e.g., B helps A in return for A having helped C; Alexander, 1987). Importantly, reciprocity requires reliable compensation, and yet it is possible for investments to be directed to ineffective members of the group who do not provide a good return on the investment (e.g., Krupp et al., 2011). The maintenance of reciprocal systems in the face of this risk has been conceptualized in various sets of models.

One of these sets can be termed “partner control” or “partner fidelity” models (e.g., Bull and Rice, 1991; Baumard et al., 2013), exemplified by situations in which two individuals are forced into interaction, often over repeated rounds. Because the social partner is predetermined and individuals can neither withdraw nor switch partners, the only recourse an individual has involves the punishment of undesirable behaviors. Here, the paradigmatic case is the iterated Prisoner’s Dilemma game, in which participants who do not cooperate with their partners can be penalized in later trials, while prosociality is matched with reciprocated prosociality (e.g., a “tit-for-tat” strategy; Axelrod and Hamilton, 1981). In this way, preventing the partner from cheating can maintain the cooperative system.

In contrast, “partner choice” models are based on the idea that individuals can be selective in their social interactions. Emphasis is placed on choosing cooperative partners and being chosen as one (e.g., Bull and Rice, 1991; Roberts, 1998). An illustrative example comes from the behavior of cleaner fish and their clients. Cleaner wrasse (*Labroides dimidiatus*) eat the ectoparasites found on the surfaces of other fish (various Australian reef fish: the “clients,”) who, in turn, benefit from the parasite removal (for review, see Bshary and Noë, 2003). Cleaner wrasse are often tolerated as they eat the ectoparasites, yet cleaners sometimes cheat by eating the client’s mucus, which is preferred over the parasites. The clients, however, find this aversive and may react in one of two ways. Sometimes clients “punish” by going on the attack, chasing, and driving the cleaners away (described as partner control), but clients may also engage in behavior that exhibits partner choice, such as swimming away and finding other cleaners. Indeed, partner choice is also evidenced by observations of clients preferably approaching cleaners who were previously observed cleaning other fish without conflict. Thus, in partner choice models, the general preference for good partners maintains reciprocity and selects for prosocial behavior within a species in the form of “social selection” (e.g., Baumard et al., 2013) and “competitive altruism” (Roberts, 1998; Barclay, 2004; Barclay and Willer, 2007).

The remainder of this paper will consider the evidence for behavior in early instances of human prosociality that is consistent with partner choice models. This is not to say that partner control models cannot describe some instances of early prosocial behavior, and there is recent informative work that may better fit that model than a partner choice model (e.g., Ingram and Bering, 2010; Vaish et al., 2011; Warneken and Tomasello, 2013). Further, for the purposes of this brief review, we do not focus on instances in which young children’s prosocial behavior may be best interpreted as the outcome of “social selection,” even though this is an important aspect of partner choice models (e.g., sharing after collaborative effort: Hamann et al., 2011; see also Warneken et al., 2011; Baumard et al., 2012; Melis et al., 2013). Instead, we will present a review of recent studies that together support the claim that early prosocial behavior is often selective in terms of recipient.

## EVIDENCE FOR PARTNER CHOICE IN EARLY PROSOCIAL DEVELOPMENT

An important prerequisite for partner choice behavior in humans would be an evaluative system that distinguishes positive interactions from negative interactions and encourages approach or other affiliative behaviors directed toward those involved in positive interactions. In this section, evidence for this evaluative system in infancy will be presented, followed by discussion of instances in which the evaluations that young children make are followed by selective engagement of prosocial behavior. Then, we will consider why, at a more proximate level, children are being selective. It is important to acknowledge, though, that selectivity in prosocial behavior – and the motivations to be selective – will become more sophisticated with age as new means of evaluation develop (e.g., Hay et al., 1991; Warneken and Tomasello, 2009, 2013; Dahl et al., 2013; see also Wynn, 2009).

### FOUNDATIONS IN INFANCY: EVALUATION OF OTHERS’ BEHAVIOR

Partner choice models present an adaptive strategy for the maintenance of reciprocity, though the existence of behaviors that support partner choice during infancy may not seem immediately adaptive. Infants cannot easily “choose” their social partners and their prosocial behavior is limited at best. Yet, arguably, the infant’s evaluation of social interaction may serve as adaptive preparation for later childhood and adulthood partner choice behavior, or even have some adaptive value during infancy, possibly as part of an attachment mechanism (i.e., serve as an “ontogenetic adaptation,” Bjorklund and Pellegrini, 2000).

In one of the first studies to examine infants’ evaluation of simple interactions among agents, Premack and Premack (1997) reported that 12-month-old infants recognize the underlying valence of helping and hindering behavior as positive and negative, respectively. Infants visually habituated to one of four interactions conveyed by animated circles: helping (one circle lifted and pushed the second, enabling it to exit a door), hindering (one circle prevented the other from exiting the door), caressing, and hitting. To the adult observer, these events can be categorized at different levels. At one level, the helping and hindering events show intention to exit a door (and, for that matter, the presence of a door), and the hitting and caressing events both depict the approach of one agent toward another in an otherwise empty scene. At another level, the events could also be categorized by valence, such that helping and caressing share a sense of positivity, and hindering and hitting share negativity. The authors proposed that infants categorized the events by valence because the infants showed dishabituation to the hitting event if habituated to either helping or caressing, but not if habituated to hindering or hitting.

The evaluation of interactions also appears to influence infants’ approach (e.g., reaching) behavior. After witnessing a wooden square enable a circle to reach the top of a hill and a triangle hinder the circle’s climb, infants as young as 10 months reach for the square more often than the triangle (Hamlin et al., 2007). Similar results are found with younger infants and when different types of helping and hindering events are depicted (Hamlin and Wynn, 2011). Further, infants appear to have similar considerations regarding others’ behavior: after being habituated to helping and hindering events involving computer-animated agents, 9- and 12-month-old infants look longer when hindered agents approach those who have hindered them in the past (Kuhlmeier et al., 2004; Hamlin et al., 2007; also see Kuhlmeier et al., 2003, for results with simple, faceless stimuli). Comparable results have been found in other laboratories and with other actions, such as harming (i.e., reaching for victims over harmful agents, Kanakogi et al., 2013) and sharing. For the latter, infants appear to be sensitive to the equal or unequal distribution of goods (Schmidt and Sommerville, 2011; Sommerville et al., 2013), and by at least 10 months, this evaluation is utilized when they subsequently consider the likelihood of another agent approaching the distributor (e.g., Geraci and Surian, 2011; Meristo and Surian, 2013). Together, these studies suggest that evaluative processes that support later selective prosociality are present within the first year of life.

### SELECTIVE PROSOCIAL BEHAVIOR BASED ON OTHERS’ HELPING, HINDERING, AND HARMING BEHAVIOR

During the second year of life and beyond, the evaluation of interactions appears to influence the selective engagement in prosocial behavior. Recent experimental paradigms have manipulated the interactions that young children witness by varying the behavioral and physical characteristics of the actors. Children’s subsequent engagement in prosocial behavior toward these individuals is then measured. The manipulated characteristics of the actors have included engagement in helping, hindering, and harming behavior, discussed here, as well as other behaviors and physical characteristics that will be discussed in later sections. Additionally, some experimental paradigms have included the child as a third-party witness of the actor’s behavior toward another actor (i.e., similar to indirect reciprocity), while others are designed with the child as a member of the interaction (i.e., similar to direct reciprocity).

Young children appear to selectively share resources with individuals who have a history of helping over individuals who have hindered. In one study (Kenward and Dahl, 2011), preschool children observed events inspired by Kuhlmeier et al. (2003) in which a puppet was trying to climb a ladder or trying to dig a hole and was helped by one character and hindered by another. Subsequently, 4.5-year-old, but not 3-year-old, children distributed resources (“biscuits”) in favor of the helper. These children also tended to justify this distribution in relation to the helper and hinderer’s previous actions. Of note, however, was that when biscuits were plentiful (e.g., eight or nine biscuits), children opted to give equal numbers to each actor, even if that meant not distributing all of the resources. Thus, factors such as an “equality bias” may eclipse selective sharing when resources are plentiful, while selectivity based on recipients’ previous behavior is observed when resources are scarce.

The selective sharing of a desired resource is also suggested in a study that presented 18- and 25-month-old children with events in which a person was either the victim of another’s harmful behavior or not a victim (Vaish et al., 2009). Children gave a balloon more often to the victim, though since this victim was not paired with the harming actor, it is unclear from this study whether children would also avoid individuals who harm others. Also, as the authors conclude, the sharing behavior may be best interpreted as the outcome of sympathy, which, while likely integral to the broader consideration of human morality, is not currently a key feature in partner choice models. A perhaps clearer example of partner choice comes from a second study by Vaish et al. (2010) which found that 3-year-old children in a forced-choice task selectively helped an actor who previously did *not* intend to harm another actor over one who did show the intention (Vaish et al., 2010). When an overtly helpful actor was paired with a neutral actor in second experimental condition, though, children were not selective in their helping behavior. Yet, notably, the “neutral” actor had previously interacted in a friendly manner with participants in a warm-up period, and thus selectivity may not have been observed simply because both actors had a history of only positive interactions.

### SELECTIVE PROSOCIAL BEHAVIOR BASED ON OTHERS’ PROVISION OF RESOURCES

Young children also appear to selectively help individuals who have shown the intention to provide resources to them. Dunfield and Kuhlmeier (2010) demonstrated that 21-month-old children selectively picked up an out-of-reach object for an individual who, in a previous interaction, intended to provide them with a desired toy over one who did not. Children selected the recipient of their helping behavior based on an actor’s positive intention even if the actor had tried but failed to deliver the toy. A subsequent experiment indicated that the children were selective even when both actors’ actions resulted in providing the toy, yet only one of the actor’s showed the overt intention to provide (i.e., the other actor’s actions were accidental).

Further evidence for selective prosocial behavior based on others’ provision of resources comes from studies in which children observe interactions between other individuals and then are given the opportunity to act. For example, Dahl et al. (2013) found that 27-month-olds were more likely to help an actor who had previously returned a desired object to another actor than one who had not returned the object. Additional analyses indicated that although 16-month-olds did not demonstrate selective helping, they also did not show the same looking time patterns as the slightly older children who did selectively help (i.e., looking longer at non-sharing interactions). It is possible that the younger participants did not understand and evaluate the interactions they observed and thus had no basis for selectivity (though see Section 3 below).

In a study with slightly older children, 3-year-olds directed a doll to give more resources to a doll that had previously given to others (Olson and Spelke, 2008). In another condition, children directed the doll to give more to someone who gave to directly to the doll than someone who gave to others, suggesting that early selective sharing behavior is constrained by a nuanced evaluation of the previously witnessed interaction and the individuals involved. Similarly, as reported in this Special Topic Volume, 15-month-old toddlers will selectively provide a resource to someone who has made equal (fair) distributions to two other people over someone who has not, but the children’s selectivity appears to be affected by the race of the distributor and recipient in relation to the participant (Burns and Sommerville, 2014).

### SELECTIVE PROSOCIAL BEHAVIOR BASED ON OTHERS’ INFORMATION SHARING

A communicative interaction often allows an individual to gain benefits that would be unavailable through individual learning alone. The provision of information can be construed as a prosocial act (e.g., Liszkowski, 2005), and by at least 3 years of age, children are more likely to apply the label “helpful” to a puppet who was willing to communicate the solution to a puzzle than to one who declared that he knew but was “not telling” (Dunfield et al., 2013). The evaluation of a communicative interaction also appears to influence selective helping behavior in young children; 3-year-olds will selectively deliver a dropped object or provide information to the informative puppet over the unwilling puppet (Dunfield et al., 2013).

In Dunfield et al. (2013), the accuracy of the puppets’ information was not manipulated (i.e., a puppet either willingly provided accurate information or simply refused to provide any information), but at least by 5 years of age, children believe that an individual who previously provided accurate information would be more likely to “share her toys” than someone who provided inaccurate information (Brosseau-Liard and Birch, 2010). This study did not examine whether children would also selectively direct their own prosocial behavior toward an accurate individual, but Brooker and Poulin-Dubois (2013) did not find evidence for greater helping behavior by 18-month-olds after an interaction with an accurate experimenter than after observing an inaccurate experimenter. However, unlike Dunfield et al. (2013), the between-subjects experimental procedure used in Brooker and Poulin-Dubois (2013) did not create a situation in which children were able to choose between these individuals. In sum, children’s assessment of an individual’s willingness to provide information does seem to influence subsequent selective helping, but future research is required to examine the influence of the accuracy of the provided information.

### SELECTIVE PROSOCIAL BEHAVIOR BASED ON GROUP MEMBERSHIP

Thus far, our discussion has focused on instances in which young children have engaged in selective prosocial behavior immediately after being directly involved in, or observing, interactions with others. A past history of interactions may also influence selective prosociality. For example, Moore (2009) found that 4–6 year-old children shared stickers (at a cost to themselves) more with friends than other familiar peers and strangers, although when there was no personal cost to providing stickers, friends and strangers were treated similarly. Friends were also favored in Olson and Spelke (2008); 3-year-olds directed a doll to give more items to her friends. However, children were only selective in the distribution when resources were scarce and they were unable to give to all of the dolls.

Young children may also engage in selective helping behavior based on defined group membership and similarity to the self, even without previous observation of social interactions. At 2.5–3 years of age, children selectively helped a puppet who was previously described as being “on their team” (group membership) or as wearing the same color shirt (similarity) over non-team members and dissimilar puppets (O’Neill and Kuhlmeier, 2013, 2014). Further suggestion comes from work by Dunham et al. (2011), in which 5-year-old children allocated resources toward in-group members even when group assignment occurred randomly and group members were previously unknown to the child (though here, children were not sharing *per se*, as they could not opt to keep the resources for themselves).

## WHY DO YOUNG CHILDREN SHOW SELECTIVITY?

The findings presented above suggest that toddlers and young children are often selective in relation to the recipient of their helping and sharing behaviors. We remain agnostic, however, as to the precise age at which prosocial partner choice can be observed. Helping behavior, such as picking up a dropped object, is observed at 14 months of age (Warneken and Tomasello, 2007), and informing is found at 12 months (Liszkowski, 2005), yet there have been no experimental attempts to examine selective helping in toddlers at this age. Some existing studies, though, do find age differences within their sample, with younger children (2 or 3 years of age) showing weaker effects of partner choice than slightly older children (e.g., Kenward and Dahl, 2011; Dahl et al., 2013). As noted above, one possible reason that a study may not find evidence for partner choice in very young children is simply that these children did not understand the social interaction that was enacted and thus could not form an evaluation on which to base their selectivity. However, unless the understanding of the interaction can be measured independently of partner choice behaviors, then the hypothesis of early selectivity is unfalsifiable. It is also important to note that even if partner choice is found in young children’s earliest instances of prosocial behavior, the mechanisms underlying selectivity may differ across development and situation.

At this point, however, the causes of selectivity are unclear. That is, while the interdisciplinary study of reciprocity has provided partner choice models that detail the important role of selectivity, the more proximate, cognitive mechanisms—particularly in early human development–have not been fully elaborated. In this section, we consider possible cognitive mechanisms underlying selectivity; however, we have opted not to discuss the mechanisms underlying selectivity based on group status in detail, as these have been well considered in social and developmental psychology literature (e.g., Billig and Tajfel, 1973; Nesdale, 2004; Tajfel and Turner, 2004; Bigler and Liben, 2007; Dunham et al., 2011).

The existence of behavior in humans and non-human animals that is consistent with partner choice models (e.g., Bshary and Noë, 2003; Warneken, 2013b) suggests both that partner choice is a fundamental system for the maintenance of reciprocity and that the proximate mechanisms that support it may range from highly constrained innate predispositions to more flexible individual and social learning processes and rational inference. An initial proposal we can make regarding early human selectivity is that at least by 3 years of age, it is *not* based on an imitative processes in which children respond to one prosocial action by re-enacting the same action; children in Dunfield et al. (2013) and Kenward and Dahl (2011) engaged in selective prosocial behaviors that differed from the previously observed behaviors.

One possibility, though, is that partner choice in young children is, in some instances, based on an expectation of reciprocity. For adults, pre-existing beliefs and the observation of behavior give rise to inferences about others’ dispositions (e.g., Kelley, 1973; Choi et al., 1999; Molden et al., 2006). It is possible that young children also engage in a process by which they form an expectation of future behavior on the part of the individual they have selected as a recipient of prosocial behavior. That is, the previous observation of an individual’s actions may lead to an attribution of a prosocial disposition, in turn leading to the assumption that the child’s own prosocial behavior is being selectively directed toward someone with whom future interactions will be generally positive (i.e., a sensitivity to the likelihood of reciprocity).

This type of attribution may be present by 3 years of age, yet further research is needed. As noted above, 3-year-old children labeled an actor who provided information as “helpful” and selectively helped that actor in return, though expectations regarding the actor’s future actions were not measured in this study (Dunfield et al., 2013). Additional initial support comes from a task in which a social partner was fixed (i.e., a task associated with partner *control* models). Warneken and Tomasello (2013) found that 3-year-old children based their sharing behavior on the sharing behavior of a fixed partner over repeated encounters (i.e., showing “contingent reciprocity”); however, there was no evidence that the actor’s behavior influenced 2-year-old children’s sharing. Thus, a preliminary proposal is that by 3 years of age, selective partner choice may also, in some situations, be based on the attribution of a prosocial disposition coupled with an expectation of reciprocity.

The attribution of a prosocial disposition (such that an individual is expected to engage in prosocial actions) may also be formed without the direct observation of prosocial behavior by that individual. For example, by at least 4 years of age, children view lucky individuals as more likely to engage in prosocial behavior (Olson et al., 2008). It is thus possible that during the first 5 years of life, children’s selective helping and sharing toward certain individuals, even in the absence of direct observation of those individuals’ prosocial actions (e.g., selective prosociality directed toward in-group members), may also be based on the attribution of a prosocial disposition. Future experimental paradigms may consider examining whether children engage in selective prosocial behavior toward individuals who demonstrate other positive traits that are not directly related to prosociality (e.g., health, strength, prestige, or intelligence).

A viable, alternative proximate cause of selective prosocial behavior is that children may simply find some individuals more positive in a general sense and engage in selective partner choice based on this positivity. That is, at some ages and in some situations, a general sense of positivity may not be translated to a dispositional attribution, yet still may lead to selectivity. When a choice is available, children may, for example, direct their own positively valenced actions toward those who have engaged in positively valenced actions themselves or those who have a positively valenced trait (e.g., member of in-group) without an explicit expectation of reciprocity. Importantly, this is not a “kill joy” explanation. Indeed, similar proposals have been made for a possible mechanism guiding partner choice based reciprocity in non-human animals (e.g., Brosnan and de Waal, 2002; Schino and Aureli, 2010). Thus, consideration of the breadth of mechanisms that can lead to effective partner choice will provide a better understanding of both the ontogeny and phylogeny of prosocial behavior.

## CONCLUSION

In sum, we suggest that many instances of early prosocial behavior produced by young children fit partner choice models of reciprocity. Recent findings suggest that early helping and sharing behaviors are often selective in terms of recipient, with selectivity based on the observation of previous actions and interactions, as well as featural characteristics of the potential recipients. The proximate causes of selective partner choice in early development require further study and may differ across age and situation, but likely candidate mechanisms range from expectations of reciprocity based on the attribution of prosocial dispositions to a more general motivation to direct positively valenced behaviors toward positively valenced individuals. The application of partner choice and partner control models to the study of childhood prosocial development—in sum, the study of reciprocity—in turn sheds light on the factors that encourage or discourage prosocial behavior in our early social interactions.

## Conflict of Interest Statement

The authors declare that the research was conducted in the absence of any commercial or financial relationships that could be construed as a potential conflict of interest.
